# Patterns of Osteoporosis Treatment Initiation Following Low‐Trauma Fracture: A Population‐Based Retrospective Cohort Study in New South Wales, Australia, Using Linked Administrative Health Data

**DOI:** 10.5694/mja2.70285

**Published:** 2026-09-01

**Authors:** Mike Lin, Huy Nguyen, Thach Tran, Robert D. Blank, Dana Bliuc, Jacqueline R. Center

**Affiliations:** ^1^ Bone Epidemiology, Clinical and Translational Science Lab Garvan Institute of Medical Research Sydney New South Wales Australia; ^2^ St Vincent's Healthcare Clinical Campus, University of New South Wales Sydney New South Wales Australia; ^3^ School of Biomedical Engineering, University of Technology Sydney Sydney New South Wales Australia; ^4^ Department of Medicine Medical College of Wisconsin Milwaukee Wisconsin USA; ^5^ North Wales Medical School, Bangor University Bangor Wales UK

**Keywords:** comorbidities, epidemiology, fracture, osteoporosis, pharmacoepidemiology, public policy

## Abstract

**Objectives:**

To characterise osteoporosis medicine prescribing patterns following low‐trauma fractures and identify predictors of early treatment initiation.

**Study Type:**

Retrospective population‐based cohort study.

**Setting:**

New South Wales, Australia, using statewide linked administrative health data.

**Participants:**

Adults aged ≥ 50 years with an incident low‐trauma fracture between January 2011 and June 2019.

**Main Outcome Measures:**

Primary outcomes were osteoporosis treatment prescribing patterns and timing of initiation after incident fracture over 3 years, categorised as early (within 12 months), late (within 1–3 years) or no initiation. Secondary analyses examined predictors of time to treatment initiation within 12 months using Fine–Gray competing‐risk regression.

**Results:**

Among 132,268 individuals with incident fractures, 63.7% (84,222) were female. Overall, 29,802 (22.5%) initiated osteoporosis medicine, whereas 102,466 (77.5%) remained untreated. Among females (mean [standard deviation] age, 77.7 [10.1] years), 20.1% initiated therapy within 12 months, 7.0% within 1–3 years, and 72.9% remained untreated. Among males (mean [standard deviation] age, 77.2 [10.0] years), the corresponding proportions were 10.8%, 3.7% and 85.6%. After accounting for the competing risk of death, treatment initiation was more likely with non‐distal fractures, older age, polypharmacy, prior steroid use, prior dual‐energy x‐ray absorptiometry and later fracture year, and less likely with greater comorbidity, rural or regional residence and prior hospitalisation for falls. The strongest associations were for hip or vertebral fracture (subdistribution hazard ratio range, 1.90–3.03) and high comorbidity burden (subdistribution hazard ratio range, 0.72–0.75). Almost one in four late initiators had a refracture before starting treatment. Denosumab rapidly replaced oral bisphosphonates as the dominant therapy over time.

**Conclusion:**

More than three‐quarters of individuals remain untreated after fracture, highlighting persistent and substantial gaps in secondary fracture prevention. Treatment initiation is strongly associated with fracture site and multimorbidity burden, and initiation rates are lower in males. Increasing reliance on denosumab underscores the need for careful long‐term treatment planning and strategies.

## Plain Language Summary

**The Known:** After a fracture, the risk of another fracture is high. Osteoporosis medicines can help reduce this risk.

**The New:** In this large study of more than 132,000 people in New South Wales, Australia, with fractures, about one in four females and one in seven males started treatment with osteoporosis medicine within 3 years. Males and people with more health problems were less likely to receive treatment. Over time, denosumab replaced oral bisphosphonates as the most common therapy.

**The Implications:** There are major gaps in post‐fracture care, highlighting missed opportunities to prevent future fractures. Earlier treatment initiation should be a priority.

## 1 Introduction

Low‐trauma fractures are common, affecting one in two women and one in four men over their lifetime [1]. They are associated with substantial morbidity, mortality and healthcare costs and indicate underlying osteoporosis [2, 3, 4].

After a fracture, individuals are at markedly increased risk of refractures, at both major osteoporotic fracture (MOF) sites—hip, vertebra, proximal humerus and wrist—and non‐MOF sites, excluding fingers, toes and skull [5, 6]. This risk is greatest in the first 1–2 years following the index fracture, a concept known as ‘imminent fracture risk’ [7, 8].

Bisphosphonates and denosumab are widely prescribed antiresorptive therapies and are subsidised through Australia's Pharmaceutical Benefits Scheme (PBS) for low‐trauma fractures [9]. Despite evidence from randomised trials that these therapies reduce future fracture risk [10], many Australians remain untreated [11, 12, 13, 14].

Multimorbidity, defined as two or more chronic conditions, affects over half of individuals with fractures and is associated with poorer outcomes, underinvestigation and undertreatment [15, 16, 17]. It increases clinical complexity, polypharmacy and treatment burden, and may shift clinical priorities away from osteoporosis care [18, 19, 20].

Understanding treatment initiation patterns following fracture is important for identifying care gaps and improving secondary fracture prevention. Population‐level data examining prescribing patterns and treatment timing remain limited.

This study aimed to characterise osteoporosis medicine prescribing patterns and treatment timing following incident fractures in New South Wales, Australia, and to identify factors associated with time to treatment initiation within 12 months.

## 2 Methods

### 2.1 Data Sources

This whole‐of‐population retrospective linked cohort study was conducted in NSW, Australia. State‐based datasets provided by the Centre for Health Record Linkage (CHeReL) included Admitted Patient Data Collection (APDC), Emergency Department Data Collection (EDDC), NSW Mental Health Ambulatory Data Collection and NSW Cancer Registry. National datasets provided by the Australian Institute of Health and Welfare (AIHW) included Medicare Consumer Directory (MCD), Medicare Benefits Schedule (MBS), PBS and National Death Index (NDI). Probabilistic record linkage was performed by CHeReL. Further details are provided in Table S1. Data were available from January 2000 to June 2022, except PBS (from 2002) and MBS, MCD and NDI (from 2005). There were no missing data for analysed variables, as all participants were Medicare‐enrolled and records were linked using unique AIHW and CHeReL identifiers. The study followed RECORD guidelines (Table S2).

### 2.2 Study Population

A closed‐source population was defined retrospectively from linked administrative health records as Medicare‐enrolled NSW residents born before 1 January 1955, and resident in NSW from 1 January 2005, with no new entrants after this date. Eligible individuals were required to have at least one emergency department presentation or hospital admission between 1 January 1 2005 and 30 June 2019. Sex was ascertained from Medicare enrolment records in the MCD and categorised as male or female.

Incident low‐trauma fractures occurring between 1 January 2011 and 30 June 2019 were identified in the APDC and EDDC using International Classification of Diseases, Tenth Revision (ICD‐10), Systematised Nomenclature of Medicine—Clinical Terms (SNOMED‐CT) and Australian Classification of Health Interventions (ACHI) procedure codes (Table S3), as previously described [21]. Individuals with any fracture recorded during the 2005–2010 lookback period (used to ensure index fractures were incident) were excluded. The index fracture was defined as the first fracture during the study period and refracture as the second fracture event. Individuals entered the analytical cohort at the time of their index fracture.

The fracture ascertainment period was selected to align with PBS availability of bisphosphonates and denosumab. Denosumab was listed from 1 December 2010 for females and 1 December 2013 for males. Individuals were followed for up to 3 years after the index fracture, until death or end of available follow‐up, whichever occurred first.

Fractures were categorised into four sites as previously described [6]: hip, vertebral, proximal (i.e., non‐hip and non‐vertebral fractures located above the knee or forearm) and distal (i.e., fractures involving the knee, forearm or regions distal to these). High‐trauma, pathological, fingers, toes and skull fractures were not counted. Further details are provided in Table S4.

### 2.3 Classification of Medication Exposure

PBS dispensing data were used to classify individuals as prior users, new users or never users. Prior users had any bone‐active medications (BAMs) dispensed in the 5 years before the index fracture, including ongoing use at fracture, and were excluded from the primary analyses. BAMs included osteoporosis medicines and agents used for bone metastases, hypercalcaemia and Paget disease (Table S5).

Among individuals without BAM use in the 5 years before fracture, new users were defined as those who initiated osteoporosis medicine within 3 years after fracture: early initiators within 12 months and late initiators between 1 and 3 years. Never users did not initiate within 3 years. The 12‐month threshold was chosen to capture treatment plausibly related to the index fracture while allowing for real‐world delays in assessment and prescribing. Initiation between 1 and 3 years was considered delayed initiation, whereas beyond 3 years was less likely to be directly attributable to the index fracture and more likely to reflect subsequent events such as refracture, interval dual‐energy x‐ray absorptiometry (DXA), new comorbidities or changes in healthcare contact.

Eligible osteoporosis medicines (Table S5) were categorised as denosumab, oral nitrogen‐containing bisphosphonates (alendronate and risedronate), intravenous zoledronic acid and other agents (calcitriol, strontium ranelate, raloxifene, etidronate, teriparatide and romosozumab).

### 2.4 Covariates

Baseline characteristics included age at fracture, fracture site, Index of Relative Socio‐economic Disadvantage (IRSD), Index of Education and Occupation (IEO) and Modified Monash Model (MMM) remoteness. IRSD and IEO scores and MMM categories were derived from Medicare enrolment postcode at time of fracture. IRSD and IEO scores were analysed in deciles, with higher deciles indicating lower relative disadvantage.

Comorbidities, Charlson Comorbidity Index (CCI) [22], Hospital Frailty Risk Score (HFRS) [23], prior hospitalisations, hospitalisation for falls and DXA use were assessed over the 5 years before fracture. Steroid exposure was defined as prednisone ≥ 5 mg within 1 year before fracture, and polypharmacy as ≥ 5 medications dispensed within 90 days before fracture. CCI scores were categorised as low (0–1), moderate (2–3) or high (≥ 4) comorbidity burden, while HFRS values were classified as low (< 5), intermediate (5–15) and high (> 15) frailty risk. Further covariate definitions are provided in Tables S6 and S7.

### 2.5 Statistical Analysis

Analyses were stratified by sex due to differing fracture and mortality risks [2, 3]. Baseline characteristics were compared using analysis of variance with Tukey correction for continuous variables and chi‐square tests for categorical variables. Cumulative incidence of treatment initiation was estimated using the Aalen–Johansen method in PROC LIFETEST (SAS version 9.4), treating death before initiation as a competing event [24].

Sex‐specific annual trends were examined for post‐fracture treatment status (early initiation, late initiation or non‐initiation) by fracture year and, among treatment initiators, medication class by year of initiation. For each calendar year, age‐specific proportions were directly standardised to the sex‐specific age distribution of the 2011 fracture cohort. Direct standardisation was used to provide comparable marginal proportions across years while accounting for changes in the age composition of the fracture cohort.

To account for the competing risk of death, Fine–Gray regression was used to examine whether age, prior hospitalisation, comorbidity burden, fracture site, remoteness, polypharmacy, prior steroid use, prior DXA use, hospitalisation for falls, fracture year and socioeconomic and educational/occupational status predicted time to treatment initiation within 12 months. The first dispensing of an osteoporosis medicine was the event of interest, and death before initiation was the competing event. Individuals who remained alive and untreated at 12 months were administratively censored. Results were reported as subdistribution hazard ratios (sHRs) with 95% confidence intervals (CIs).

The functional form of continuous covariates was assessed graphically and by comparing linear specifications with more flexible or categorical alternatives. No important departures from linearity were identified for variables retained as linear terms [25]. Age at fracture was modelled per 5‐year increase, and IRSD and IEO scores were modelled per one‐decile increase. Fracture year was modelled categorically, with 2011 as the reference year, to account for secular changes in prescribing. Due to collinearity between the CCI scores and HFRS values, a separate multivariable model was fitted using HFRS in place of CCI.

Analyses were conducted using SAS version 9.4 (SAS Institute). A two‐sided *p* value of < 0.05 was considered statistically significant.

### 2.6 Ethics Statement

The study was approved by the AIHW (EO2020/5/1192), and data were deidentified.

## 3 Results

**FIGURE 1:**
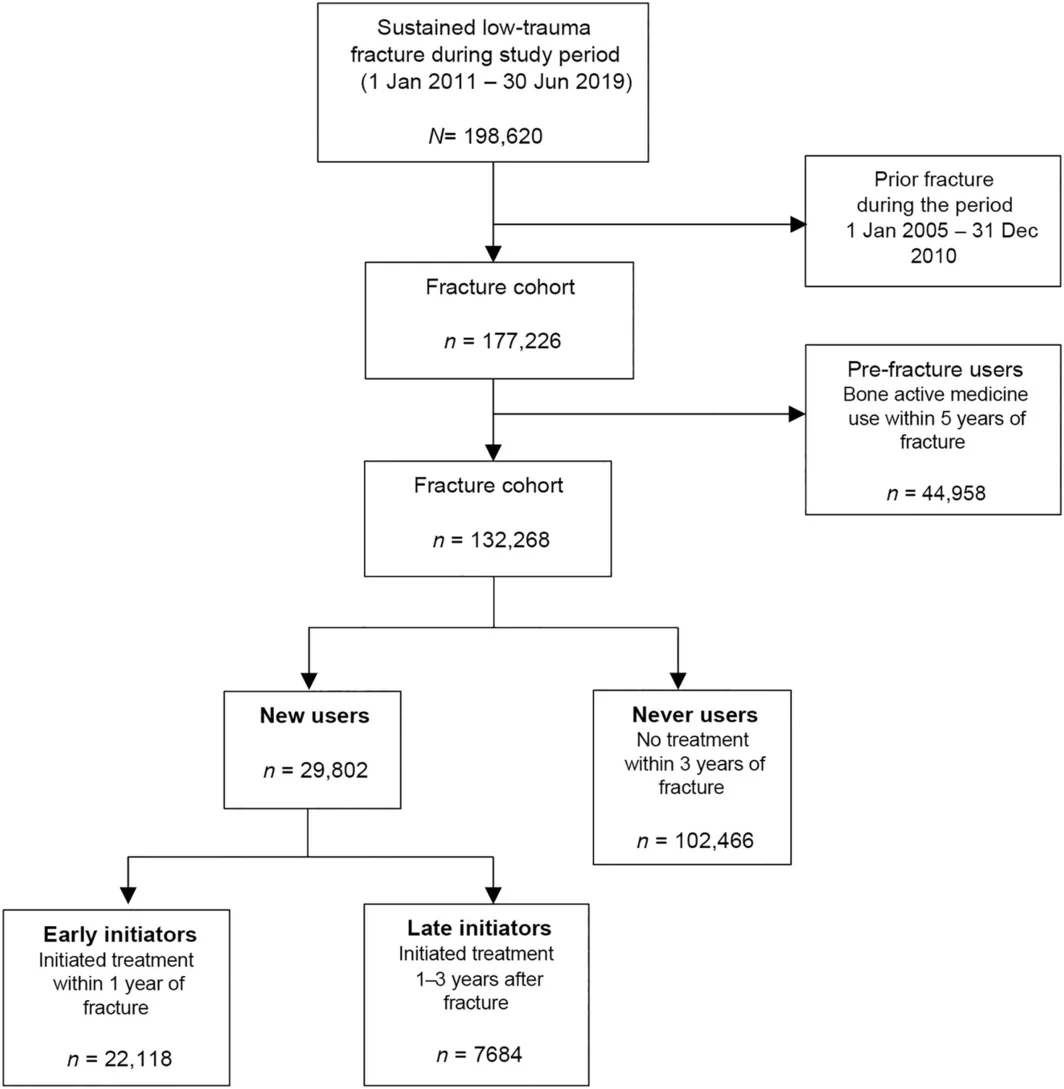
Study flowchart showing cohort selection and classification of pre‐fracture users, new users and never users of osteoporosis medicines following low‐trauma fracture.

The study flowchart is shown in Figure 1. Of 198,620 individuals with fractures, 21,394 had prior fractures, and 44,958 were prior users and were excluded. Among the remaining 132,268 eligible individuals with incident fractures, 84,222 (63.7%) were female, 29,802 (22.5%) were new users, and 102,466 (77.5%) were never users.

Baseline characteristics are summarised in Table 1. Among women (mean [standard deviation] age, 77.7 [10.1] years), 16,952 (20.1%) initiated treatment within 12 months, 5913 (7.0%) initiated treatment between 1 and 3 years, while 61,357 (72.9%) received no post‐fracture treatment. Uptake was lower among males (mean [standard deviation] age 77.2 [10.0] years); 5166 (10.8%) initiated treatment within 12 months, 1771 (3.7%) between 1 and 3 years, and 41,109 (85.6%) received no treatment.

**TABLE 1:** Baseline characteristics of new and never users stratified by sex.

Baseline characteristic	Female (*n* = 84,222)^a^			Male (*n* = 48,046)^a^		
	New users		Never users (*n* = 61,357; 72.9%)	New users		Never users (*n* = 41,109; 85.6%)
	Early initiators (*n* = 16,952; 20.1%)	Late initiators (*n* = 5913; 7.0%)		Early initiators (*n* = 5166; 10.8%)	Late initiators (*n* = 1771; 3.7%)	
Age at fracture, years, mean (SD)	77.5 (9.3)	75.8 (8.7)	75.9 (10.7)	79.8 (8.6)	77.7 (8.5)	76.0 (10.2)
Number of hospitalisations in prior 5 years, mean (SD)	2.6 (3.3)	2.7 (3.8)	2.5 (3.4)	4.1 (4.5)	3.9 (4.3)	3.6 (4.3)
IRSD decile,^b^ median (IQR)	6 (4–9)	5 (3–9)	5 (3–9)	5 (3–9)	5 (3–9)	5 (3–8)
IEO decile,^b^ median (IQR)	6 (4–9)	6 (3–9)	6 (3–9)	6 (4–9)	6 (4–9)	6 (3–9)
Remoteness, *n* (%)						
Metropolitan	12,011 (70.9%)	4120 (69.7%)	41,243 (67.2%)	3775 (73.1%)	1238 (69.9%)	27,418 (66.7%)
Regional	1997 (11.8%)	681 (11.5%)	7571 (12.3%)	545 (10.5%)	227 (12.8%)	4953 (12.0%)
Rural	2944 (17.4%)	1112 (18.8%)	12,543 (20.4%)	846 (16.4%)	306 (17.3%)	8738 (21.3%)
Comorbidity burden, *n* (%)						
Low (CCI 0–1)	13,904 (82.0%)	4888 (82.7%)	49,572 (80.8%)	3422 (66.2%)	1206 (68.1%)	28,377 (69.0%)
Moderate (CCI 2–3)	2193 (12.9%)	750 (12.7%)	7941 (12.9%)	1158 (22.4%)	373 (21.1%)	7817 (19.0%)
High (CCI > 4)	855 (5.0%)	275 (4.7%)	3844 (6.3%)	586 (11.3%)	192 (10.8%)	4915 (12.0%)
Hospital Frailty Risk Score, *n* (%)						
Low (< 5)	12,282 (72.5%)	4456 (75.4%)	43,917 (71.6%)	2895 (56.0%)	1109 (62.6%)	25,604 (62.3%)
Intermediate (5–15)	2965 (17.5%)	973 (16.5%)	9922 (16.2%)	1281 (24.8%)	410 (23.2%)	8449 (20.6%)
High (> 15)	1705 (10.1%)	484 (8.2%)	7518 (12.3%)	990 (19.2%)	252 (14.2%)	7056 (17.2%)
Fracture site, *n* (%)						
Hip	4687 (27.6%)	1003 (17.0%)	11,217 (18.3%)	2041 (39.5%)	427 (24.1%)	8088 (19.7%)
Spine	1451 (8.6%)	370 (6.3%)	2927 (4.8%)	854 (16.5%)	217 (12.3%)	3558 (8.7%)
Proximal	4136 (24.4%)	1606 (27.2%)	15,880 (25.9%)	1296 (25.1%)	576 (32.5%)	14,715 (35.8%)
Distal	6678 (39.4%)	2934 (49.6%)	31,333 (51.1%)	975 (18.9%)	551 (31.1%)	14,748 (35.9%)
Prior hospitalisation for fall, *n* (%)	2162 (12.8%)	645 (10.9%)	8498 (13.9%)	1098 (21.3%)	296 (16.7%)	7561 (18.4%)
Prior DXA imaging, *n* (%)	4825 (28.5%)	1802 (30.5%)	11,900 (19.4%)	931 (18.0%)	314 (17.7%)	4182 (10.2%)
Prior steroids, *n* (%)	1267 (7.5%)	401 (6.8%)	3377 (5.5%)	491 (9.5%)	152 (8.6%)	2586 (6.3%)
Polypharmacy (≥ 5 medications), *n* (%)	5757 (34.0%)	1951 (33.0%)	19,128 (31.2%)	2366 (45.8%)	780 (44.0%)	15,007 (36.5%)
Comorbidities, *n* (%)						
Anxiety/depression	862 (5.1%)	275 (4.7%)	3595 (5.9%)	256 (5.0%)	78 (4.4%)	2375 (5.8%)
Cancer	1044 (6.2%)	342 (5.8%)	4001 (6.5%)	660 (12.8%)	211 (11.9%)	5111 (12.4%)
Congestive heart failure	875 (5.2%)	274 (4.6%)	3819 (6.2%)	509 (9.9%)	151 (8.5%)	3946 (9.6%)
Chronic kidney disease						
Stage 1–3	388 (2.3%)	141 (2.4%)	1664 (2.7%)	333 (6.4%)	109 (6.2%)	2375 (5.8%)
Stage 4–5 or dialysis	314 (1.9%)	115 (1.9%)	1251 (2.0%)	248 (4.8%)	106 (6.0%)	1482 (3.6%)
Chronic obstructive pulmonary disease	893 (5.3%)	307 (5.2%)	3241 (5.3%)	483 (9.3%)	146 (8.2%)	3366 (8.2%)
Dementia	583 (3.4%)	98 (1.7%)	3799 (6.2%)	266 (5.1%)	58 (3.3%)	2889 (7.0%)
Diabetes	2080 (12.3%)	694 (11.7%)	7581 (12.4%)	1073 (20.8%)	363 (20.5%)	7470 (18.2%)
Gastro‐malabsorption	125 (0.7%)	57 (1.0%)	345 (0.6%)	37 (0.7%)	16 (0.9%)	236 (0.6%)
Gastro‐oesophageal reflux disease	1138 (6.7%)	389 (6.6%)	3763 (6.1%)	327 (6.3%)	115 (6.5%)	2431 (5.9%)
Hyperlipidaemia	389 (2.3%)	134 (2.3%)	1422 (2.3%)	180 (3.5%)	65 (3.7%)	1401 (3.4%)
Hyperparathyroidism	52 (0.3%)	23 (0.4%)	152 (0.2%)	13 (0.3%)	3 (0.2%)	60 (0.1%)
Hypertension	2946 (17.4%)	951 (16.1%)	10,723 (17.5%)	1407 (27.2%)	438 (24.7%)	9262 (22.5%)
Hyperthyroidism	190 (1.1%)	62 (1.0%)	857 (1.4%)	43 (0.8%)	16 (0.9%)	330 (0.8%)
Ischaemic heart disease	1014 (6.0%)	369 (6.2%)	3536 (5.8%)	625 (12.1%)	233 (13.2%)	4710 (11.5%)
Chronic liver disease	144 (0.8%)	69 (1.2%)	639 (1.0%)	110 (2.1%)	41 (2.3%)	971 (2.4%)
Osteoporosis	432 (2.5%)	122 (2.1%)	1441 (2.3%)	321 (6.2%)	111 (6.3%)	2437 (5.9%)
Rheumatoid arthritis	112 (0.7%)	35 (0.6%)	238 (0.4%)	32 (0.6%)	6 (0.3%)	109 (0.3%)
Stroke	827 (4.9%)	246 (4.2%)	3109 (5.1%)	514 (9.9%)	136 (7.7%)	3355 (8.2%)

Abbreviations: CCI, Charlson Comorbidity Index; DXA, dual‐energy x‐ray absorptiometry; IEO, Index of Education and Occupation; IQR, interquartile range; IRSD, Index of Relative Socio‐economic Disadvantage; SD, standard deviation.

^a^ Totals may not always sum to 100% due to rounding.

^b^ IRSD and IEO are reported as deciles. Higher decile scores indicate lower socioeconomic disadvantage and greater educational and occupational advantage, respectively.

In both sexes, early initiators were older than late initiators and never users (pairwise *p* < 0.01). Distal fractures were most common among never users, whereas hip and vertebral fractures were more frequent among early initiators. Never users had greater comorbidity burden and were more likely to live in rural areas (*p* < 0.001 for all).

Compared with females, males had a higher comorbidity burden, greater frailty risk and higher healthcare utilisation, including more prior hospitalisations, hospitalisation for falls and polypharmacy (*p* < 0.001 for all).

### 3.1 Medicine Choice Among Early Initiators

Medicine choice differed by sex (Table 2). Among females, denosumab was most frequently prescribed (53.3%), followed by oral nitrogen‐containing bisphosphonates (34.3%), intravenous zoledronic acid (6.7%) and other osteoporosis medicines (5.8%). Within the other category, most prescriptions were for strontium ranelate (72.6%), calcitriol (19.7%) and raloxifene (7.7%). Among males, denosumab (45.0%) and oral nitrogen‐containing bisphosphonates (41.7%) were prescribed at similar rates, while intravenous zoledronic acid (8.0%) and other medications (5.3%) were less common. Within the other category, calcitriol accounted for most prescriptions (65.1%), followed by strontium ranelate (34.5%) and raloxifene (0.4%). No osteoanabolic therapies were prescribed for either sex.

**TABLE 2:** Baseline characteristics of early initiators by drug choice.

Baseline characteristic	New users—early initiators^a^							
	Female (*n* = 16,952)				Male (*n* = 5166)			
	Dmab (*n* = 9027; 53.3%)	Oral N‐BP (*n* = 5812; 34.3%)	Intravenous ZOL (*n* = 1138; 6.7%)	Other drugs^b^ (*n* = 975; 5.8%)	Dmab (*n* = 2325; 45.0%)	Oral N‐BP (*n* = 2152; 41.7%)	Intravenous ZOL (*n* = 414; 8.0%)	Other drugs^c^ (*n* = 275; 5.3%)
Age at fracture, years, mean (SD)	78.0 (8.9)	77.1 (9.5)	74.6 (8.9)	77.8 (10.4)	80.7 (8.2)	79.3 (8.7)	77.0 (8.9)	80.1 (8.9)
Number of hospitalisations in prior 5 years, mean (SD)	2.8 (3.4)	2.3 (3.0)	2.8 (3.3)	3.1 (3.9)	4.2 (4.4)	3.8 (4.2)	3.8 (4.3)	6.1 (6.8)
IRSD decile, median (IQR)	6 (4–9)	5 (3–8)	8 (5–10)	5 (3–8)	6 (4–9)	5 (3–8)	7 (4–10)	5 (3–9)
IEO decile, median (IQR)	6 (4–9)	6 (4–9)	9 (5–10)	6 (3–9)	6 (4–9)	6 (3–9)	8 (4–10)	4 (2–8)
Remoteness, *n* (%)								
Metropolitan	6391 (70.8%)	4063 (69.9%)	898 (78.9%)	659 (67.6%)	1693 (72.8%)	1573 (73.1%)	327 (79.0%)	182 (66.2%)
Regional	1086 (12.0%)	678 (11.7%)	103 (9.1%)	130 (13.3%)	270 (11.6%)	193 (9.0%)	40 (9.7%)	42 (15.3%)
Rural	1550 (17.2%)	1071 (18.4%)	137 (12.0%)	186 (19.1%)	362 (15.6%)	386 (17.9%)	47 (11.4%)	51 (18.5%)
Comorbidity burden, *n* (%)								
Low (CCI 0–1)	7340 (81.3%)	4888 (84.1%)	942 (82.8%)	734 (75.3%)	1515 (65.2%)	1500 (69.7%)	294 (71.0%)	113 (41.1%)
Moderate (CCI 2–3)	1209 (13.4%)	699 (12.0%)	141 (12.4%)	144 (14.8%)	551 (23.7%)	449 (20.9%)	88 (21.3%)	70 (25.5%)
High (CCI ≥ 4)	478 (5.3%)	225 (3.9%)	55 (4.8%)	97 (9.9%)	259 (11.1%)	203 (9.4%)	32 (7.7%)	92 (33.5%)
Hospital Frailty Risk Score, *n* (%)								
Low (< 5)	6376 (70.6%)	4374 (75.3%)	858 (75.4%)	674 (69.1%)	1246 (53.6%)	1264 (58.7%)	274 (66.2%)	111 (40.4%)
Intermediate (5–15)	1682 (18.6%)	905 (15.6%)	193 (17.0%)	185 (19.0%)	581 (25.0%)	524 (24.3%)	86 (20.8%)	90 (32.7%)
High (> 15)	969 (10.7%)	533 (9.2%)	87 (7.6%)	116 (11.9%)	498 (21.4%)	364 (16.9%)	54 (13.0%)	74 (26.9%)
Fracture site, *n* (%)								
Hip	2402 (26.6%)	1733 (29.8%)	236 (20.7%)	316 (32.4%)	926 (39.8%)	866 (40.2%)	152 (36.7%)	97 (35.3%)
Spine	768 (8.5%)	495 (8.5%)	103 (9.1%)	85 (8.7%)	391 (16.8%)	355 (16.5%)	65 (15.7%)	43 (15.6%)
Proximal	2260 (25.0%)	1369 (23.6%)	277 (24.3%)	230 (23.6%)	593 (25.5%)	547 (25.4%)	85 (20.5%)	71 (25.8%)
Distal	3597 (39.8%)	2215 (38.1%)	522 (45.9%)	344 (35.3%)	415 (17.8%)	384 (17.8%)	112 (27.1%)	64 (23.3%)
Prior hospitalisation for fall, *n* (%)	1206 (13.4%)	715 (12.3%)	122 (10.7%)	119 (12.2%)	553 (23.8%)	429 (19.9%)	57 (13.8%)	59 (21.5%)
Prior DXA imaging, *n* (%)	2793 (30.9%)	1403 (24.1%)	391 (34.4%)	239 (24.5%)	487 (20.9%)	305 (14.2%)	100 (24.2%)	39 (14.2%)
Prior steroids, *n* (%)	707 (7.8%)	405 (7.0%)	95 (8.3%)	60 (6.2%)	221 (9.5%)	197 (9.2%)	42 (10.1%)	31 (11.3%)
Polypharmacy (≥ 5 medications), *n* (%)	3169 (35.1%)	1858 (32.0%)	364 (32.0%)	366 (37.5%)	1097 (47.2%)	949 (44.1%)	173 (41.8%)	147 (53.5%)

Abbreviations: CCI, Charlson Comorbidity Index; Dmab, denosumab; DXA, dual‐energy x‐ray absorptiometry; IEO, Index of Education and Occupation; IQR, interquartile range; IRSD, Index of Relative Socio‐economic Disadvantage; N‐BP, nitrogen containing bisphosphonate; SD, standard deviation; ZOL, zoledronic acid.

^a^ Totals may not always sum to 100% due to rounding.

^b^ Other drugs among female early initiators included calcitriol (192 [19.7%]), strontium ranelate (708 [72.6%]) and raloxifene (75 [7.7%]).

^c^ Other drugs among male early initiators included calcitriol (179 [65.1%]), strontium ranelate (95 [34.5%]) and raloxifene (1 [0.4%]).

Baseline characteristics differed by drug class (Table S8). Intravenous zoledronic acid users were younger and more likely to have prior DXA imaging, lower socioeconomic and education and occupational disadvantage, and metropolitan residence than users of other therapies (*p* < 0.001 for all). Denosumab and oral nitrogen‐containing bisphosphonate users had broadly similar characteristics, although chronic kidney disease was more common among denosumab users (*p* < 0.001). By contrast, calcitriol users had substantially more cardiometabolic conditions, including chronic kidney disease, diabetes, hypertension, ischaemic heart disease and congestive heart failure, and higher comorbidity and frailty burden (*p* < 0.001 for all).

### 3.2 Timing of Treatment Initiation

**FIGURE 2:**
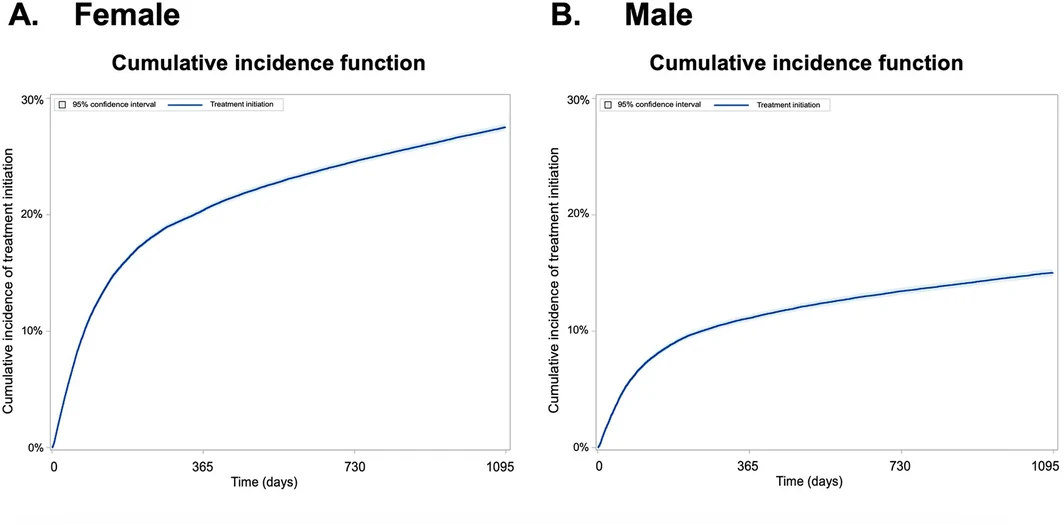
Cumulative incidence curve for osteoporosis treatment initiation. Cumulative incidence curves for osteoporosis treatment initiation following low‐trauma fracture in (A) females and (B) males, accounting for death as a competing event. The *y*‐axes represent cumulative probability of treatment initiation, expressed as a proportion. Shaded bands represent pointwise 95% confidence intervals.

Figure 2 presents the cumulative incidence function for treatment initiation with death treated as a competing event. The corresponding numbers of initiators and deaths are presented in Table 3. Cumulative incidence of treatment initiation within 12 months was 20.4% (95% CI, 20.1%–20.7%) in females (Figure 2A) and 11.1% (95% CI, 10.9%–11.4%) in males (Figure 2B). By 3 years, this increased to 27.5% (95% CI, 27.2%–27.8%) and 15.0% (95% CI, 14.7%–15.4%), respectively. The cumulative incidence of death before treatment initiation at 3 years was 16.5% (95% CI, 16.3%–16.8%) in females and 28.5% (95% CI, 28.1%–28.9%) in males.

**TABLE 3:** Cumulative incidence of osteoporosis treatment initiation and death before initiation after fracture.^a^

Time after fracture (days)	Initiated treatment, *n*	Treatment initiation CIF, % (95% CI)	Died before initiation, *n*	Death before initiation CIF, % (95% CI)
Female				
365	16,952	20.4% (20.1%–20.7%)	7447	8.8% (8.7%–9.0%)
730	20,697	24.6% (24.3%–24.9%)	10,859	12.9% (12.7%–13.1%)
1095	22,865	27.5% (27.2%–27.8%)	13,912	16.5% (16.3%–16.8%)
Male				
365	5166	11.1% (10.9%–11.4%)	7985	16.6% (16.3%–17.0%)
730	6220	13.4% (13.1%–13.8%)	11,115	23.1% (22.8%–23.5%)
1095	6937	15.0% (14.7%–15.4%)	13,699	28.5% (28.1%–28.9%)

Abbreviations: CI, confidence interval; CIF, cumulative incidence function.

^a^ Treatment initiation was the event of interest, and death before treatment initiation was treated as a competing event. Counts are crude cumulative numbers by each time point; percentages are CIF estimates.

Among individuals initiating treatment within 3 years, median (interquartile range) time to treatment initiation was 130 (50–378) days in females and 120 (45–375) days in males. Refracture before treatment initiation was more common among late initiators than early initiators: 24.2% versus 5.7% in females and 23.9% versus 6.1% in males. This suggests late initiators may be triggered by refracture rather than the index fracture.

### 3.3 Temporal Trends in Treatment Initiation

**FIGURE 3:**
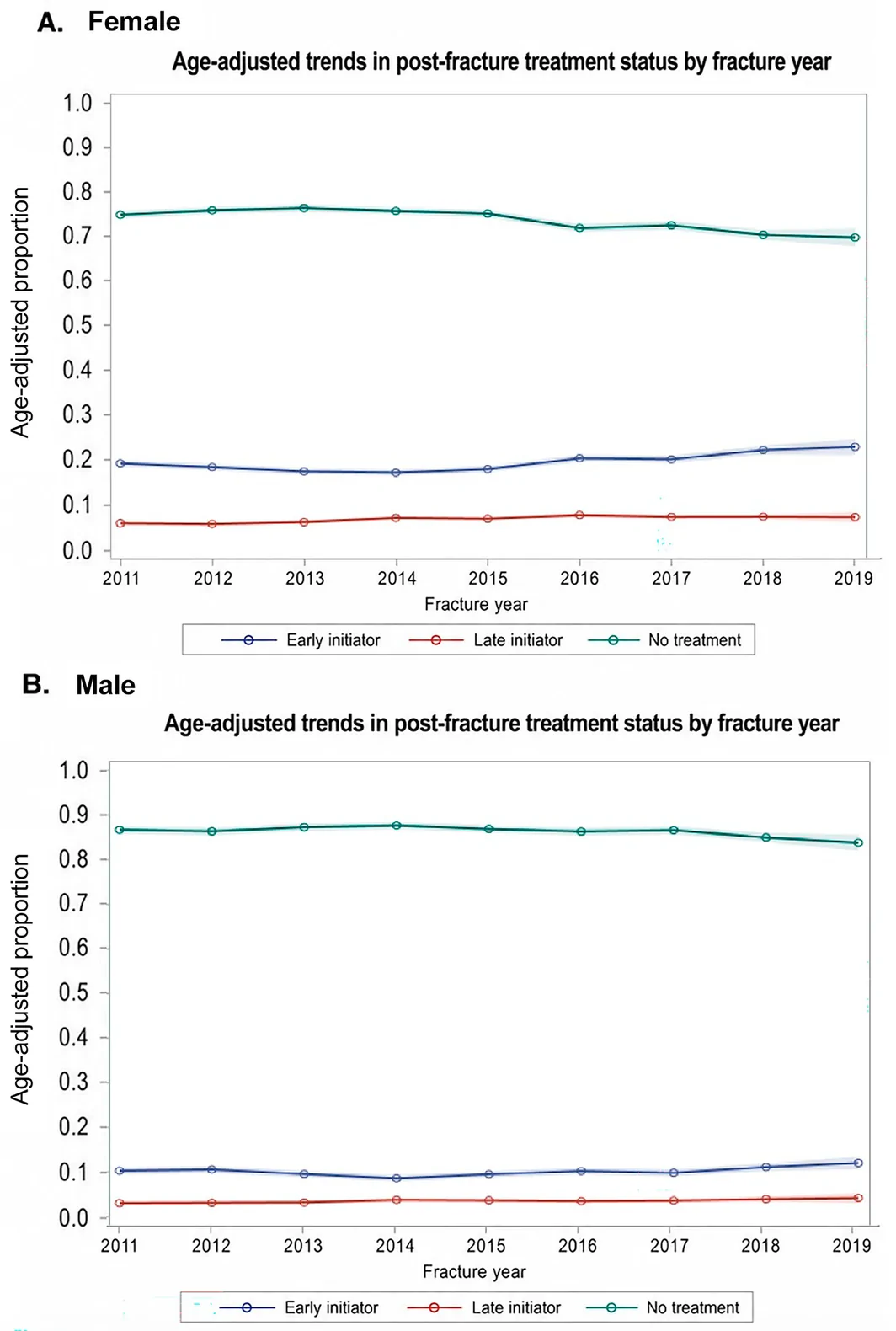
Age‐adjusted trends in early initiation, late initiation and no treatment by fracture year in (A) females and (B) males. Shaded bands represent pointwise 95% confidence intervals for the age‐adjusted proportions.

Treatment initiation improved only marginally over the study period and remained low overall (Figure 3). After accounting for changes in the age distribution over time, early initiation among females (Figure 3A) increased from 19.2% in 2011 to 22.9% in early 2019, while the untreated proportion declined from 74.9% to 69.9%. Among males (Figure 3B), early initiation increased from 10.3% in 2011 to 12.0% in early 2019, while the untreated proportion declined from 86.7% to 83.8%. The annual number of fracture events remained stable between 2011 and 2018, ranging from 9600 to 10,300 among females and 5300 to 5800 among males.

### 3.4 Temporal Trends in Drug Choice

**FIGURE 4:**
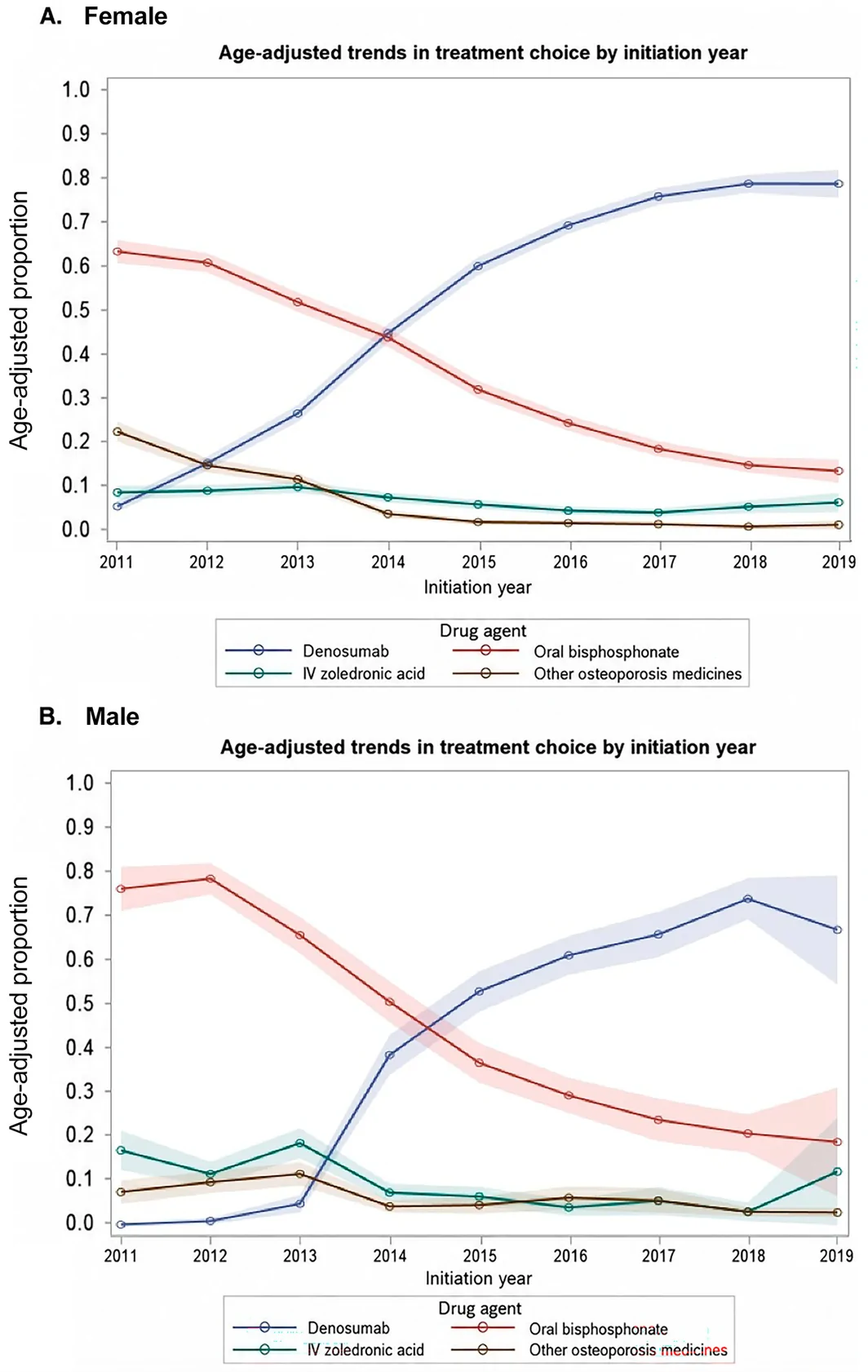
Age‐adjusted distribution of osteoporosis medication classes among treatment initiators following low‐trauma fracture by year of treatment initiation in (A) females and (B) males. Shaded bands represent pointwise 95% confidence intervals for the age‐adjusted proportions. IV, intravenous.

Age‐adjusted prescribing patterns among treatment initiators changed substantially over time (Figure 4). Among females (Figure 4A), oral nitrogen‐containing bisphosphonates were the most common therapy in 2011 (63.0%), but denosumab became dominant by 2014. By early 2019, 78.2% of females initiating therapy received denosumab, while oral nitrogen‐containing bisphosphonate use declined to 13.6%. Intravenous zoledronic acid use remained low and stable, and use of other medicines declined after 2013. A similar pattern was observed among males (Figure 4B). Oral nitrogen‐containing bisphosphonates predominated in 2011 (75.5%), but denosumab became the most commonly used therapy by 2015 and accounted for 66.3% of initiations by early 2019. Use of intravenous zoledronic acid and other medicines remained uncommon.

### 3.5 Predictors of Early Treatment Initiation

Table 4 presents sex‐specific sHRs from Fine–Gray competing‐risk regression models of time to treatment initiation within 12 months following fracture. Greater comorbidity burden was consistently associated with a lower subdistribution hazard of treatment initiation. Compared with individuals with low comorbidity burden (CCI 0–1), those with high comorbidity burden (CCI ≥ 4) had a lower subdistribution hazard of initiation in both females (sHR, 0.75 [95% CI, 0.70–0.81]) and males (sHR, 0.72 [95% CI, 0.66–0.80]).

**TABLE 4:** Baseline predictors of osteoporosis treatment initiation within 12 months after low‐trauma fracture.^a^

Variable	Female multivariable sHR (95% CI)	Male multivariable sHR (95% CI)
Age at fracture, per 5‐year increase	1.02 (1.01–1.03)^b^	1.07 (1.06–1.09)^b^
Prior hospitalisation	1.00 (1.00–1.01)	1.01 (1.00–1.02)
Comorbidity burden		
Low (CCI 0–1)	Reference	Reference
Medium (CCI 2–3)	0.89 (0.85–0.94)^b^	0.97 (0.90–1.04)
High (CCI ≥ 4)	0.75 (0.70–0.81)^b^	0.72 (0.66–0.80)^b^
Fracture site		
Distal	Reference	Reference
Hip	1.90 (1.82–1.98)^b^	3.03 (2.78–3.31)^b^
Vertebral	2.12 (2.00–2.25)^b^	2.92 (2.65–3.22)^b^
Proximal	1.20 (1.16–1.25)^b^	1.26 (1.16–1.37)^b^
Remoteness		
Metropolitan	Reference	Reference
Regional	0.96 (0.91–0.99)^b^	0.81 (0.74–0.89)^b^
Rural	0.87 (0.84–0.91)^b^	0.73 (0.67–0.79)^b^
Polypharmacy		
No	Reference	Reference
Yes	1.04 (1.01–1.08)^b^	1.22 (1.15–1.30)^b^
Prior steroids		
No	Reference	Reference
Yes	1.22 (1.15–1.29)^b^	1.25 (1.13–1.37)^b^
Prior DXA imaging		
No	Reference	Reference
Yes	1.48 (1.43–1.53)^b^	1.67 (1.56–1.80)^b^
Prior hospitalisation for fall		
No	Reference	Reference
Yes	0.81 (0.77–0.85)^b^	0.86 (0.80–0.92)^b^
IRSD, per one‐decile increase	0.97 (0.96–0.98)^b^	0.96 (0.94–0.98)^b^
IEO, per one‐decile increase	1.04 (1.03–1.06)^b^	1.05 (1.02–1.07)^b^
Fracture year		
2011	Reference	Reference
2012	0.96 (0.90–1.02)	1.05 (0.93–1.18)
2013	0.90 (0.85–0.96)^b^	0.94 (0.83–1.05)
2014	0.88 (0.83–0.94)^b^	0.83 (0.74–0.94)^b^
2015	0.93 (0.88–1.00)	0.95 (0.84–1.06)
2016	1.09 (1.02–1.16)^b^	1.02 (0.91–1.15)
2017	1.07 (1.01–1.14)^b^	0.99 (0.89–1.11)
2018	1.20 (1.13–1.28)^b^	1.14 (1.02–1.27)^b^
2019	1.28 (1.19–1.37)^b^	1.27 (1.11–1.44)^b^

Abbreviations: CCI, Charlson Comorbidity Index; CI, confidence interval; DXA, dual‐energy x‐ray absorptiometry; IEO, Index of Education and Occupation; IRSD, Index of Relative Socio‐economic Disadvantage; sHR, subdistribution hazard ratio.

^a^ The sHR and 95% CIs values were estimated using Fine–Gray competing‐risk regression. An sHR of < 1 indicates a lower cumulative incidence of treatment initiation within 12 months whereas an sHR of > 1 indicates a higher cumulative incidence of treatment initiation. The event of interest was first osteoporosis treatment supply within 12 months of the index fracture, with death before treatment initiation treated as a competing event. Individuals who remained alive and untreated at 12 months were censored at 12 months. Models included all variables shown and calendar year of fracture.

^b^ Statistically significant sHR.

Fracture site was strongly associated with treatment initiation. Compared with distal fractures, hip fractures were associated with a higher subdistribution hazard of initiation in females (sHR, 1.90 [95% CI, 1.82–1.99]) and males (sHR, 3.04 [95% CI, 2.79–3.32]). Vertebral fractures showed a similar pattern, with larger hazards in males (sHR, 2.93 [95% CI, 2.66–3.23]) than in females (sHR, 2.12 [95% CI, 2.00–2.26]). Associations for proximal fractures were more modest.

Polypharmacy, prior DXA use and steroid exposure were associated with a higher subdistribution hazard of initiation, whereas a history of hospitalisation for falls and rural or regional residence were associated with a lower subdistribution hazard in both sexes. Findings were similar when HFRS replaced CCI: compared with low frailty risk, high frailty risk was associated with a lower subdistribution hazard of initiation in females (sHR, 0.64 [95% CI, 0.60–0.69]) and males (sHR, 0.70 [95% CI, 0.63–0.77]).

## 4 Discussion

In this large cohort study of over 132,000 adults in NSW, Australia, with fractures requiring hospital or emergency admission, several important findings emerged. First, nearly three‐quarters of females and more than four‐fifths of males remained untreated following fracture, highlighting a substantial gap and gender disparity in secondary fracture prevention. Due to the 2005–2010 lookback period applied to define incident index fractures, the cohort likely underestimates the total number of low‐trauma fractures occurring. The population‐level burden of fracture may therefore be greater than reflected in the analytical cohort. Second, treatment initiation was strongly influenced by fracture site, with hip and vertebral fractures more likely to prompt therapy than distal fractures. Third, greater multimorbidity was associated with lower likelihood of treatment. Finally, although overall treatment rates changed little over time, drug choice changed markedly, with denosumab rapidly replacing oral bisphosphonates as the most common therapy.

These findings are concerning given the period of imminent fracture risk—the risk of subsequent fracture is highest in the first 1–2 years after the index fracture [7]. Early treatment is critical, as antiresorptive treatments reduce fracture risk and may mitigate this high‐risk period [26]. The low treatment rates in our cohort are consistent with those seen in other studies from Australia and studies from the United States, Korea and Spain, where only 10%–30% of patients receive osteoporosis medicines following fracture [12, 15, 27, 28, 29]. Despite increasing awareness and implementation of fracture liaison services, treatment remains suboptimal [30]. Furthermore, nearly one‐quarter of late initiators sustained a refracture before starting treatment, suggesting that treatment was often prompted by refracture rather than the index fracture.

Fracture site was strongly associated with treatment initiation. Hip and vertebral fractures are widely recognised as osteoporotic fractures and carry substantial morbidity and mortality, which may prompt more active management [31]. In contrast, non‐MOF fractures such as ankle, lower leg and rib fractures may be under‐recognised despite evidence that they reflect skeletal fragility and predict subsequent fracture and premature mortality [2, 6, 32]. The lower cumulative incidence of treatment initiation following distal fractures therefore presents an important missed opportunity.

Multimorbidity was also strongly associated with treatment initiation. Individuals with high comorbidity burden (CCI ≥ 4) had a lower cumulative incidence of treatment initiation. By contrast, polypharmacy was associated with higher treatment initiation, suggesting that these measures capture different dimensions of complexity: multimorbidity may reduce prioritisation of osteoporosis treatment amid competing health priorities, whereas polypharmacy may mark greater healthcare contact and prescribing opportunity [33, 34]. Treatment may also be modified rather than omitted in medically complex patients. Calcitriol users had higher frailty and cardiometabolic comorbidity burden, suggesting preferential use in more complex patients where conventional antiresorptive therapies may be used more cautiously, despite calcitriol's weaker antifracture efficacy [35].

Sex differences were evident throughout. The cumulative incidence of treatment initiation was lower in males than in females, consistent with previous literature [36, 37]. Osteoporosis has historically been perceived as a condition affecting females, which contributes to under‐recognition and undertreatment in males. However, fracture‐related morbidity and mortality are often higher in men [38], highlighting the need to improve management in this population.

Although overall treatment rates changed little, prescribing patterns shifted substantially. Denosumab rapidly became the dominant post‐fracture therapy, replacing oral bisphosphonates, which is consistent with Australian general practice trends [14]. Likely contributors include improved adherence with 6‐monthly dosing, clinician familiarity and accumulating evidence for antifracture efficacy [39, 40]. However, denosumab introduces discontinuation challenges, including rebound bone loss and increased vertebral fracture risk. As denosumab use increases, careful treatment sequencing and discontinuation planning will become increasingly important [41]. Since November 2024, romosozumab has become PBS subsidised for first‐line treatment of severe osteoporosis with high fracture risk [9]. This does not alter our interpretation of undertreatment, but highlights the need for risk‐stratified treatment selection and sequencing.

### 4.1 Limitations

Several limitations should be acknowledged. First, our 12‐month definition of early initiation was generous compared with recent Australian standards recommending pharmacotherapy within 20 weeks of fracture [42], and may have underestimated delayed initiation. However, this threshold was chosen pragmatically to account for real‐world delays in assessment and prescribing. Second, administrative data rely on coded diagnoses and may misclassify or under‐ascertain comorbidities [43]. In addition, redundant EDDC SNOMED‐CT codes may have reduced precision, although ICD‐10 and ACHI procedural codes were also used for fracture ascertainment [44]. Third, PBS data may not have captured all osteoporosis‐related medication use, including private prescriptions, under‐copayment dispensing before April 2012 and medicines administered during hospital admissions. This may have caused under‐ascertainment of some covariates, such as exposure to prednisone and use of intravenous zoledronic acid that was administered through hospital fracture liaison services. However, substantial undercapture of osteoporosis treatment is unlikely because antiresorptive therapies are PBS subsidised as first‐line treatment after fracture, and the proportion of late initiators remained low even after accounting for possible inpatient intravenous zoledronic acid use. Finally, lifestyle factors and DXA‐derived bone mineral density data were unavailable. Nevertheless, low‐trauma fractures in adults aged ≥ 50 years are widely considered osteoporotic and warrant treatment [9].

## 5 Conclusion

This population‐based cohort study highlights major gaps in secondary fracture prevention in Australia, with more than three‐quarters of patients untreated after fracture. Treatment initiation was particularly low among males, multimorbid individuals and those with distal fractures. Nearly one‐quarter of treatment initiation after the first post‐fracture year followed a refracture. Denosumab overtook bisphosphonates as the predominant therapy. These findings highlight the need for better systems of care to support timely treatment during the period of imminent fracture risk.

## Author Contributions

Conceptualisation: Mike Lin, Dana Bliuc, Jacqueline R. Center. Data curation: all authors. Formal analysis: all authors. Funding acquisition: Dana Bliuc, Jacqueline R. Center. Investigation: all authors. Methodology: Mike Lin, Dana Bliuc, Jacqueline R. Center. Project administration: Mike Lin, Dana Bliuc, Jacqueline R. Center. Resources: Mike Lin, Dana Bliuc, Jacqueline R. Center. Supervision: Dana Bliuc, Jacqueline R. Center. Writing – original draft: Mike Lin. Writing – review and editing: all authors.

## Funding

This work was supported by an Amgen Foundation grant (Competitive Bone Grant Prolia BCGP‐06 to Dana Bliuc), a National Health and Medical Research Council grant (1108886 to Jacqueline R. Center) and a Medical Research Future Fund grant (1137462 to Jacqueline R. Center). The funders had no role in study design, data collection and analysis, decision to publish or preparation of the manuscript.

## Disclosure

Not commissioned; externally peer reviewed.

## Conflicts of Interest

The authors declare no conflicts of interest.

## Supporting information


**Table S1:** Linked data sources used in the study.
**Table S2:** The RECORD statement—checklist of items, extended from the STROBE statement, that should be reported in observational studies using routinely collected health data.
**Table S3:** List of ICD‐10, Systematised Nomenclature of Medicine—Clinical Terminology (SNOMED‐CT) and Australian Classification of Health Intervention (ACHI) codes used to define fractures.
**Table S4:** Fracture definitions.
**Table S5:** Medications, PBS codes and dosing schedules.
**Table S6:** List of ICD‐10 codes used to define Charlson Comorbidity Index, Hospital Frailty Risk Score and other chronic diseases of interest.
**Table S7:** Explanation of the covariates used in the mortality and refracture risk analyses.
**Table S8:** Baseline characteristics of early initiators by drug choice.

## Data Availability

The datasets used and analysed during this study are available from Secure Unified Research Environment (SURE), which is independently managed by the Sax Institute. Per the data use agreement between the authors and SURE, the deposition of data into publicly available repositories is not allowed.
