# Supplementary material for: Patterns of Osteoporosis Treatment Initiation Following Low‐Trauma Fracture: A Population‐Based Retrospective Cohort Study in New South Wales, Australia, Using Linked Administrative Health Data
Source: Med J Aust. 2026 Sep 1;224(9):e70285. doi: 10.5694/mja2.70285 (PMC13531723; doi:10.5694/mja2.70285)
Supplement: Supplementary file 1 [file MJA2-224-0-s001.pdf]

The Medical Journal of Australia

Supporting Information

Supplementary material

This appendix was part of the submitted manuscript and has been peer reviewed. It is posted as supplied by the authors.

Appendix to: M. Lin, H. Nguyen, T. Tran, et al. Patterns of osteoporosis treatment initiation following low-trauma fracture: a population-based retrospective cohort study in New South Wales, Australia, using linked administrative health data. *Medical Journal of Australia* 2026; doi: 10.5694/mja2.70285.

Table S1: Linked data sources used in the study

Data source	Origin	Data collection period	Information captured	Study use
Admitted Patient Data Collection (APDC)	CHeReL	2000 - 2022	Public and private hospital admissions in New South Wales, Australia	Identification of fractures, hospitalisation, comorbidities and hospitalised falls
Emergency Department Data Collection (EDDC)	CHeReL	2000 - 2022	Emergency department presentations	Identification of fractures and emergency presentations
NSW Mental Health Ambulatory Data Collection (MHADC)	CHeReL	2000 - 2022	Ambulatory mental health service contacts	Identification of mental health diagnoses
NSW Cancer Registry	CHeReL	2000 - 2022	Reportable cancer diagnoses	Identification of cancer diagnoses
Medicare Consumer Directory (MCD)	AIHW	2005 – 2022	Medicare enrolment information, including recorded sex and residential postcode	Identification of cohort eligibility, sex, residential postcode for socioeconomic indices and remoteness
Medicare Benefits Schedule (MBS)	AIHW	2005 – 2022	Medicare-subsidised medical services	Identification of prior dual-energy x-ray absorptiometry (DXA) imaging
Pharmaceutical Benefits Scheme (PBS)	AIHW	2002 – 2022	Dispensed subsidised medications	Identification of osteoporosis medicine dispensing, corticosteroid exposure and polypharmacy
National Death Index (NDI)	AIHW	2005 - 2022	Date of death	Ascertainment of mortality

Probabilistic record linkage was performed by The Centre for Health Record Linkage. The linkage process is designed to minimise false-positive links, with an estimated false-positive rate of approximately 5 per 1,000 linked records (https://www.cherel.org.au/quality-assurance). A list of validation studies is also provided (https://www.cherel.org.au/validation-studies). Abbreviations: CheReL, The Centre for Health Record Linkage; AIHW, Australian Institute of Health and Welfare.

Table S2: The RECORD statement – checklist of items, extended from the STROBE statement, that should be reported in observational studies using routinely collected health data

	Item No.	STROBE items	Location in manuscript where items are reported	RECORD items	Location in manuscript where items are reported
**Title and abstract**					
	1	(a) Indicate the study's design with a commonly used term in the title or the abstract (b) Provide in the abstract an informative and balanced summary of what was done and what was found		RECORD 1.1: The type of data used should be specified in the title or abstract. When possible, the name of the databases used should be included.<br><br>RECORD 1.2: If applicable, the geographic region and timeframe within which the study took place should be reported in the title or abstract.<br><br>RECORD 1.3: If linkage between databases was conducted for the study, this should be clearly stated in the title or abstract.	RECORD 1.1: Abstract - Study Type; Methods - Data sources.<br><br>RECORD 1.2: Title; Abstract - Participants.<br><br>RECORD 1.3: Abstract - Study Type; Methods - Data sources.
**Introduction**					
Background rationale	2	Explain the scientific background and rationale for the investigation being reported			Introduction.
Objectives	3	State specific objectives, including any prespecified hypotheses			Introduction - final paragraph (study aims).
**Methods**					
Study Design	4	Present key elements of study design early in the paper			Abstract - Study Type; Methods - Data sources.
Setting	5	Describe the setting, locations, and relevant dates, including periods of recruitment, exposure, follow-up, and data collection			Methods - Data sources and Study population; Supplementary Table S1.
Participants	6	*(a) Cohort study* - Give the eligibility criteria, and the sources and methods of selection of participants. Describe methods of follow-up *Case-control study* - Give the eligibility criteria, and the sources and		RECORD 6.1: The methods of study population selection (such as codes or algorithms used to identify subjects) should be listed in detail. If this is not possible, an explanation should be provided.	RECORD 6.1: Methods - Study population and Classification of medicine exposure; Supplementa

		methods of case ascertainment and control selection. Give the rationale for the choice of cases and controls<br>*Cross-sectional study* - Give the eligibility criteria, and the sources and methods of selection of participants<br><br>*(b) Cohort study* - For matched studies, give matching criteria and number of exposed and unexposed<br>*Case-control study* - For matched studies, give matching criteria and the number of controls per case		RECORD 6.2: Any validation studies of the codes or algorithms used to select the population should be referenced. If validation was conducted for this study and not published elsewhere, detailed methods and results should be provided.<br><br>RECORD 6.3: If the study involved linkage of databases, consider use of a flow diagram or other graphical display to demonstrate the data linkage process, including the number of individuals with linked data at each stage.	ry Tables S3-S5.<br><br>RECORD 6.2: Methods - Study population; Supplementary Table S1 (CheReL quality-assurance and validation links).<br><br>RECORD 6.3: Methods - Data sources; Supplementary Table S1; Figure 1 (cohort selection).
Variables	7	Clearly define all outcomes, exposures, predictors, potential confounders, and effect modifiers. Give diagnostic criteria, if applicable.		RECORD 7.1: A complete list of codes and algorithms used to classify exposures, outcomes, confounders, and effect modifiers should be provided. If these cannot be reported, an explanation should be provided.	Methods - Study population, Classification of medicine exposure and Covariates; Supplementary Tables S3-S7.
Data sources/ measurement	8	For each variable of interest, give sources of data and details of methods of assessment (measurement). Describe comparability of assessment methods if there is more than one group			Methods - Data sources, Study population, Classification of medicine exposure and Covariates; Supplementary Tables S1,S3-S7.
Bias	9	Describe any efforts to address potential sources of bias			Methods - Study population and Statistical analysis; Discussion - Limitations.
Study size	10	Explain how the study size was arrived at			N/A - descriptive, population-based study

					including all eligible individuals during the study period.
Quantitative variables	11	Explain how quantitative variables were handled in the analyses. If applicable, describe which groupings were chosen, and why			Methods - Covariates and Statistical analysis; Table 1 footnote; Supplementary Table S7.
Statistical methods	12	(a) Describe all statistical methods, including those used to control for confounding<br>(b) Describe any methods used to examine subgroups and interactions<br>(c) Explain how missing data were addressed<br>(d) *Cohort study* - If applicable, explain how loss to follow-up was addressed<br>*Case-control study* - If applicable, explain how matching of cases and controls was addressed<br>*Cross-sectional study* - If applicable, describe analytical methods taking account of sampling strategy<br>(e) Describe any sensitivity analyses			(a) Methods - Covariates and Statistical analysis; Table 4 footnote.<br>(b) Methods - Statistical analysis (sex-stratified analyses, annual trends and alternative HFRS model).<br>(c) Methods - Data sources (no missing data for the primary outcome).<br>(d) Methods - Study population (follow-up to death or study end) and Statistical analysis (death treated as a competing event).<br>(e) Methods - Statistical analysis; Results - Predictors of early treatment initiation

					(HFRS model replacing CCI).
Data access and cleaning methods		..		RECORD 12.1: Authors should describe the extent to which the investigators had access to the database population used to create the study population.<br><br>RECORD 12.2: Authors should provide information on the data cleaning methods used in the study.	RECORD 12.1: Acknowledgments - Author Contributions (full access to the study data).<br><br>RECORD 12.2: Methods - Study population; Supplementary Table S4 (fracture filtering and event-classification rules).
Linkage		..		RECORD 12.3: State whether the study included person-level, institutional-level, or other data linkage across two or more databases. The methods of linkage and methods of linkage quality evaluation should be provided.	Methods - Data sources; Supplementary Table S1 (person-level probabilistic linkage, estimated false-positive rate and validation links).
**Results**					
Participants	13	(a) Report the numbers of individuals at each stage of the study (*e.g.*, numbers potentially eligible, examined for eligibility, confirmed eligible, included in the study, completing follow-up, and analysed)<br>(b) Give reasons for non-participation at each stage.<br>(c) Consider use of a flow diagram		RECORD 13.1: Describe in detail the selection of the persons included in the study (*i.e.,* study population selection) including filtering based on data quality, data availability and linkage. The selection of included persons can be described in the text and/or by means of the study flow diagram.	Results - first paragraph; Figure 1.
Descriptive data	14	(a) Give characteristics of study participants (*e.g.*, demographic, clinical, social) and information			(a) Results - Tables 1 and 2; Supplementary Table S8.

		on exposures and potential confounders<br>(b) Indicate the number of participants with missing data for each variable of interest<br>(c) *Cohort study* - summarise follow-up time (*e.g.*, average and total amount)			(b) Methods - there were no missing data for the primary outcome.<br>(c) Results - Timing of treatment initiation; Table 3.
Outcome data	15	*Cohort study* - Report numbers of outcome events or summary measures over time<br>*Case-control study* - Report numbers in each exposure category, or summary measures of exposure<br>*Cross-sectional study* - Report numbers of outcome events or summary measures			Results - first paragraph, Timing of treatment initiation and Temporal trends; Table 3; Figures 2-4.
Main results	16	(a) Give unadjusted estimates and, if applicable, confounder-adjusted estimates and their precision (e.g., 95% confidence interval). Make clear which confounders were adjusted for and why they were included<br>(b) Report category boundaries when continuous variables were categorized<br>(c) If relevant, consider translating estimates of relative risk into absolute risk for a meaningful time period			(a) Methods - Covariates and Statistical analysis; Results - Predictors of early treatment initiation; Table 4 and footnote.<br>(b) Methods - Covariates and Statistical analysis; Tables 1 and 2; Supplementary Table S7.<br>(c) Results - Timing of treatment initiation; Table 3 (absolute cumulative incidence at 1, 2 and 3 years).
Other analyses	17	Report other analyses done—e.g., analyses of			Results - Medicine

		subgroups and interactions, and sensitivity analyses			choice, Temporal trends and Predictors of early treatment initiation; Supplementary Table S8; alternative HFRS model reported in the Predictors section.
**Discussion**					
Key results	18	Summarise key results with reference to study objectives			Discussion - first paragraph.
Limitations	19	Discuss limitations of the study, taking into account sources of potential bias or imprecision. Discuss both direction and magnitude of any potential bias		RECORD 19.1: Discuss the implications of using data that were not created or collected to answer the specific research question(s). Include discussion of misclassification bias, unmeasured confounding, missing data, and changing eligibility over time, as they pertain to the study being reported.	Discussion - Limitations.
Interpretation	20	Give a cautious overall interpretation of results considering objectives, limitations, multiplicity of analyses, results from similar studies, and other relevant evidence			Discussion; Conclusion.
Generalisability	21	Discuss the generalisability (external validity) of the study results			Discussion – Limitations; Conclusion (NSW/Australian context).
**Other Information**					
Funding	22	Give the source of funding and the role of the funders for the present study and, if applicable, for the original study on which the present article is based			Acknowledgments - Funding.
Accessibility of protocol, raw data, and		..		RECORD 22.1: Authors should provide information on how to access any supplemental information such as the study	Acknowledgements - Data availability.

programmin g code				protocol, raw data, or programming code.	

*Reference: Benchimol EI, Smeeth L, Guttmann A, Harron K, Moher D, Petersen I, Sørensen HT, von Elm E, Langan SM, the RECORD Working Committee. The REporting of studies Conducted using Observational Routinely-collected health Data (RECORD) Statement. *PLoS Medicine* 2015; in press.

*Checklist is protected under Creative Commons Attribution (CC BY) license.

Table S3: List of ICD-10, Systematised Nomenclature of Medicine – Clinical Terminology (SNOMED-CT) and Australian Classification of Health Intervention (ACHI) codes used to define fractures

Fractures	ICD-10 codes	SNOMED-CT codes
Hip	S72.0- S72.2	47519, 49318, 1705000, 5913000, 12746007, 20100009, 20854409, 20855004, 22802006, 26938002, 30489009, 41191003, 52450003, 62125005, 66483005, 71620000, 84320000, 87173003, 89820008, 127287001, 208521006, 208523009, 208524003, 208526001, 208528000, 208529008, 208530003, 208531004, 208534007, 208536009, 208537000, 208540000, 208541001, 208542008, 208543003, 208548007, 208551000, 208552007, 208555009, 208557001, 208558006, 208559003, 208562000, 209334003, 209336001, 209338000, 263225007, 263226008, 263227004, 263228009, 263229001, 263231005, 272411004, 275338001, 275339009, 275340006, 275341005, 275342003, 275343008, 301034006, 302034009, 361118003, 361119006, 426382004, 427688003, 431031001, 445875009, 445899009
Vertebrae	S22.0, S22.1, S32.0, S32.2, S32.7, S32.8, T08.x	47684-00, 47690-00, 47696-00, 47699-00, 47699-01, 47699-02, 8303001, 11782000, 13498003, 15724005, 17463000, 20763004, 21573009, 26907003, 28425007, 28753006, 42157000, 42942008, 50448004, 52622006, 72346008, 78687007, 83635002, 87804006, 89825003, 111622009, 125606003, 125608002, 129241004, 129242006, 178674000, 178675004, 178676003, 178677007, 178685003, 178686002, 202831001, 203450003, 203451004, 203452006, 207895002, 207896001, 207897005, 207898000, 207899008, 207900003, 207901004, 207902006, 207903001, 207904007, 207905008, 207906009, 207907000, 207908005, 207909002, 207921003, 207922005, 207923000, 207924006, 207925007, 207926008, 207927004, 207928009, 207929001, 207930006, 207931005, 207932003, 207932003, 207933008, 207934002, 207935001, 207938004, 207939007, 207940009, 207941008, 207942001, 207943006, 207944000, 207945004, 207949005, 207950005, 207951009, 207952002, 207956004, 207957008, 207958003, 207959006, 207960001, 207961002, 207964005, 207965006, 207966007, 207967003, 207968008, 207972007, 207983003, 207984009, 207986006, 208215006, 240198002, 263065002, 263068000, 263069008, 263071008, 263072001, 263178004, 263180005, 269061001, 269062008, 269063003, 269064009, 269065005, 269067002, 269068007, 269069004, 269071004, 269072006, 269073001, 269076009, 269077000, 274153008, 274154002, 274155001, 274156000, 281907005, 281908000, 281909008, 281910003, 281911004, 281912006, 281913001, 281914007, 281915008, 281916009, 281917000, 281918005, 281919002, 281920008, 281921007, 281922000, 281923005, 281924004, 281925003, 281926002, 281927006, 281929009, 281930004, 281931000, 281932007, 281933002, 281934008, 281935009, 281936005, 281937001, 281938006, 281940001, 281941002, 281981009, 307137009, 311806008, 311815001, 311890007, 311891006, 314667001, 426646004,

		430948002, 445734009, 473435002, 473436001, 473438000
Proximal fractures:		
Pelvis	S32.3-S32.5	47489, 47480-00, 47483-00, 47486-00, 47495-00, 47498-00, 47501-00, 7687006, 13695006, 15474008, 20817406, 30060003, 33118001, 36127009, 36417005, 46272008, 60214001, 64455005, 76911006, 77493009, 77598002, 125871005, 125872003, 207977001, 207978006, 207979003, 207980000, 207981001, 208145002, 208147005, 208148000, 208149008, 208150008, 208151007, 208155003, 208156002, 208158001, 208159009, 208160004, 208165009, 208166005, 208168006, 208172005, 208173000, 208181004, 208182006, 208183001, 208184007, 208185008, 208192003, 208193008, 208194002, 208197009, 208198004, 209390009, 209395004, 209400003, 209405008, 263067005, 263095005, 263096006, 263216001, 263217005, 263218000, 263219008, 263220002, 263221003, 281995007, 281996008, 281997004, 281998009, 281999001, 282001006, 282003009, 287076006, 288280006, 301032005, 301033000, 426679004, 426962008, 428723000, 429766001, 441499007, 441881007
Femur	S72.3-S72.9	47516-01, 47528-00, 47528-01, 47531-00, 6628008, 19652000, 25415003, 26442006, 28576007, 30905007, 43975501, 54441004, 66926007, 82640004, 208578003, 208579006, 208580009, 208581008, 208582001, 208587007, 208588002, 208589005, 208590001, 208591002, 263232003, 263233008, 263234002, 263236000, 272300006, 429342005, 431030000, 439217005, 439425009, 439937002, 440627004, 440708003, 447394009
Humerus	S42.x	47423, 47426, 47429, 47432, 47444, 47447, 47450, 47451, 23741005, 42636007, 43295006, 52784000, 54567007, 66308002, 72435006, 73156009, 73244003, 86844009, 89294002, 90235006, 90358006, 122773008, 127286005, 208241000, 208244008, 208250003, 208251004, 208253001, 208254007, 208255008, 208256009, 208257000, 209236003, 209238002, 209243009, 209246001, 209248000, 209249008, 263075004, 263077007, 263098007, 263191003, 281961008, 281962001, 281963006, 287073003, 413875004, 416645004, 416833005, 417039001, 440438008, 440553003, 441309001
Rib	S22.3-S22.4	1261007, 3291007, 10568008, 12204004, 12204004, 14675005, 14950009, 17633000, 20121009, 20274005, 22000002, 28081005, 31693001, 33737001, 39335003, 44039000, 44932007, 45356009, 45910007, 46043003, 47402002, 51760006, 57577003, 58154005, 60667009, 68650003, 69029002, 77546004, 79546008, 89636007, 90863004, 176635002, 208136003, 440143005
Clavicle	S42.0	47462, 47465, 1658003, 5642004, 11163803, 33173003, 48561006, 58150001, 71039006, 87376003, 88196000, 111637008, 263099004
Distal fractures:		
Forearm	S52.x	1431, 47378, 47381, 47384, 47385, 47386, 48387, 649002, 2295008, 3228009, 12676007, 19259001, 23813004, 25529004, 27094009, 27094009, 28078000, 33041006, 33141009, 34578006, 35442005, 37449000, 39993000, 42760000, 42945005, 53627009, 53792000, 54556006, 58722007, 58722007, 65775005, 65966004, 66246007, 69427008, 71139009, 71555008, 72497001, 75857000, 78490005, 78980006, 79858001, 80411001, 81966000, 91296001, 91419009, 111640008, 111641007, 123618009, 123619001, 123971006, 123972004, 123973009, 208295005, 208303009, 208304003, 208309008, 208312006, 208318005, 208322000, 208325003, 208336005, 208337001,

		208341002, 208344005, 208926006, 209256002, 209262007, 263101006, 263101006, 263195007, 263195007, 263196008, 263197004, 263198009, 263203001, 263204007, 263206009, 263207000, 269083002, 271576001, 281968002, 281969005, 281970006, 287074009, 287074009, 307172007, 359823001, 359823001, 390986009, 426467005, 429655000, 429655000
Lower leg	S82.2-S82.8	1507, 1508, 1509, 1510, 3990005, 9344009, 20433007, 21867001, 22234005, 23900009, 26311301, 28012007, 28359007, 31978002, 47848000, 59556008, 59639009, 67394003, 75591007, 77803008, 79670000, 111646002, 208596007, 208597003, 208598008, 208600002, 208601003, 208610006, 208611005, 208614002, 208615001, 208619007, 208620001, 208621002, 208622009, 208623004, 208624005, 208625006, 208627003, 208631009, 208632002, 209339008, 209340005, 209342002, 209344001, 209346004, 263089009, 263090000, 263237009, 263238004, 263239007, 271577005, 281533006, 281843000, 307178006, 413877007, 414293001, 414943006, 428020005, 428151000, 428256003, 428257007, 428797006, 428798001, 442205007, 442538002, 446979005, 446980008, 447017008, 447139008, 447395005
Knee	S82.0	1495, 1500, 1506, 1521, 20934005, 42802005, 111643005, 111643005, 208596007, 208597003, 208598008, 208600002, 208601003, 208602005, 208602005, 208603000, 208603000, 208604006, 208604006, 208605007, 208605007, 209339008, 209341009, 209342002, 209343007, 209343007, 209344001, 209345000, 209345000, 209346004, 209347008, 209347008, 263089009, 263090000, 263113001, 307178006, 428019004, 428019004, 428151000
Ankle	S82.5-S82.6	3381004, 4673003, 47596-00, 47600-00, 47600-01, 47603-00, 47603-01, 47609-02, 47609-03, 47612-02, 47612-04, 47612-05, 47615-02, 47615-03, 47618-02, 47618-03, 47618-06, 47618-07, 6698000, 90558-00, 15385006, 16114001, 25899002, 26176009, 26324000, 26909008, 34268009, 34432001, 35899005, 42188001, 48187004, 76926009, 87905008, 90338005, 123975002, 208634001, 208635000, 208636004, 208637008, 208638003, 208639006, 208657007, 208658002, 208659005, 208660000, 208662008, 208663003, 208664009, 208665005, 208666006, 208667002, 208668007, 208669004, 208684008, 208685009, 208686005, 208696001, 208697005, 208698000, 209348003, 209349006, 209350006, 209351005, 209352003, 263091001, 263115008, 263240009, 263242001, 263246003, 278537006, 281531008, 281532001, 281535004, 281972003, 281973008, 281974002, 281975001, 281976000, 281977009, 281978004, 281979007, 281980005, 287078007, 307727005, 308153009, 315643003, 429664005, 439365009, 445410003, 446981007
Foot	S92.0-S92.3, S92.7, S92.9	47609, 47612, 47615, 47618, 47621, 47624, 47630, 47636, 47639, 47612-02, 47612-03, 47618-04, 47618-05, 4977005, 9808005, 11254008, 15574005, 18724009, 23020007, 24212007, 24948002, 26646003, 31103006, 36924003, 42306005, 46020007, 47233002, 54013005, 54075005, 56128006, 57772006, 64665009, 66973001, 67422008, 70204006, 71790003, 73015009, 75308009, 77505009, 77551005, 111647006, 208677000, 208678005, 208687001, 208688006, 208689003, 208690007, 208699008, 208700009, 208701008, 208702001, 208703006, 209353008, 209354002, 209355001, 209356000, 209357009, 209362005, 209363000, 209364006, 209365007, 209370000, 209371001, 209372008, 209373003, 209379004, 209380001, 209381002, 209382009, 263116009, 263245004, 263248002, 263249005, 263250005,

		263251009, 263252002, 263253007, 269100000, 274132004, 275094008, 281536003, 302037002, 307372000, 342070009, 367527001, 424648000, 424817005, 428018007, 428258002, 429339004, 441490006, 441516001, 441558003, 441564005, 447138000
Hand	S62.0-S62.4, S62.8	1453, 1456, 1370007, 3331008, 4349002, 7551007, 9275003, 20511007, 46422008, 52694000, 54641008, 55716004, 55874001, 78292000, 208385000, 208390002, 208393000, 208394006, 208396008, 208397004, 208399001, 208400008, 208401007, 208402000, 208403005, 208404004, 208413002, 208416005, 208417001, 208418006, 208419003, 208420009, 208421008, 208422001, 208423006, 208424000, 208428002, 209312000, 263106001, 263209002, 263210007, 263211006, 276813006, 287075005, 307173002, 340036002, 439992006, 440558007, 472856002, 722240003

Table S4: Fracture definitions

Variable	Definition
Proximal fractures	Non-hip and non-vertebral fractures above the knee or forearm
Distal fractures	Fractures involving the knee, forearm or anatomical regions distal to these
High-trauma definition	Fractures identified using ICD-10 trauma-related codes (V00-V99, W10-W17, W20-W64, X80-X82, Y35-Y36) or fracture events with more than three fracture sites recorded on the same date
Multiple fractures on same date (3 or less)	When multiple eligible fractures occurred within the same event, a single fracture site was assigned using a prespecified anatomical hierarchy, with the most proximal site retained

Table S5: Medications, PBS codes and dosing schedules

**Osteoporosis medicines**	
Medication Name	PBS item code
Denosumab	
Denosumab 60mg	5457F
Intravenous bisphosphonates	
Zoledronic acid 5mg (Aclasta)	9288W
Zoledronic acid 5mg (Ostira)	10555M
Oral nitrogen-containing bisphosphonates	
Alendronate 70mg	8511Y
Alendronate 70mg	13499L
Alendronate 70mg	2215N
Alendronate 10mg	8102K
Alendronate 70mg + Vit D3 70mcg	14003B
Alendronate 70mg + Vit D3 70mcg	9012H
Alendronate 70mg + Vit D3 70mcg	2194L
Alendronate 70mg + Vit D3 70mcg	2491B
Alendronate 70mg + Vit D3 140mcg	9183H
Alendronate 70mg + Vit D3 140mcg	2224C
Alendronate 70mg + Vit D3 140mcg	13835E
Alendronate 70mg + Ca 500mg + Vit D3 140mcg	9351E
Alendronate 70mg + Ca 500mg + Vit D3 140mcg	2273P
Risedronate 150mg	13488X
Risedronate 150mg	9391G
Risedronate 35mg	13459J

Risedronate 35mg	14197F
Risedronate 35mg	4444X
Risedronate 35mg	8621R
Risedronate EC 35mg	13364J
Risedronate EC 35mg	14198G
Risedronate EC 35mg	2191H
Risedronate EC 35mg	8972F
Risedronate 5mg	13360E
Risedronate 5mg	14209W
Risedronate 5mg	4443W
Risedronate 5mg	8481J
Risedronate 35mg + Ca 500mg	8899J
Risedronate 35mg + Ca 500mg	4059P
Risedronate 35mg + Ca 500mg	2220W
Risedronate 35mg + Ca 500mg	8973G
Risedronate 35mg + Ca 1000mg + Vit D 22mcg	9147K
Risedronate 35mg + Ca 1000mg + Vit D 22mcg	2254P
Risedronate 35mg + Ca 1000mg + Vit D 22mcg	8974H
Risedronate 35mg + Ca 1000mg + Vit D 22mcg	4380M
Other osteoporosis medicines	
Teriparatide 20mcg	14093R
Teriparatide 20mcg	14482F
Teriparatide 20mcg	9411H
Teriparatide 20mcg	12670W
Romosozumab 120mg	12301K
Disodium etidronate 200mg + Ca 500mg	8056B
Disodium etidronate 200mg	2920Q
Calcitriol 0.25mcg	2502Q
Calcitriol 0.25mcg	13457G
Strontium ranelate 2g	3036T

Raloxifene 60mg	8363E
Raloxifene 60mg	13426P

**Other bone active medicines for non-osteoporosis indications (e.g. Pagets, breast cancer, bone metastases, hypercalcaemia)**	
Medication name	PBS item code
Denosumab	5110Y
Denosumab	10061M
Zoledronic acid	9350D
Zoledronic acid	6371H
Zoledronic acid	9653C
Zoledronic acid	13772W
Zoledronic acid	13773X
Zoledronic acid	10561W
Zoledronic acid	10542W
Zoledronic acid	10548E
Zoledronic acid	10554L
Zoledronic acid	10561W
Zoledronic acid	11405G
Zoledronic acid	11388J
Zoledronic acid	10571J
Tiludronate	08267D
Alendronate	8090T
Risedronate	8482K
Ibandronate	9357L
Ibandronic acid	5750P
Ibandronic acid	9619G
Clodronic acid	8132B
Clodronic acid	8265B
Pamidronate	6286W
Pamidronate	8461H

Pamidronate	5667G
Pamidronate	8208B
Pamidronate	8209C
Pamidronate	5668H
Pamidronate	5669J
Pamidronate	5670K
Pamidronate	5701C
Pamidronate	5702D
Pamidronate	5703E
Pamidronate	6223M
Pamidronate	6279L
Pamidronate	6287X
Pamidronate	6288Y
Pamidronate	6289B
Pamidronate	6290C
Pamidronate	8209C
Pamidronate	8462J
Pamidronate	8463K
Pamidronate	6343W
Burosumab	13155J

Table S6: List of ICD-10 codes used to define Charlson Comorbidity Index, Hospital Frailty Risk Score and other chronic diseases of interest

**Comorbidities**	**ICD-10 codes**
**Charlson Comorbidity Index**	
Myocardial infarction	I21, I22, I25.2
Congestive heart failure	I09.9, I11.0, I13.0, I13.2, I25.5, I42.0, I42.5 - I42.9, I43, I50, P29.0
Peripheral vascular disease	I70, I71, I73.1, I73.8, I73.9, I77.1, I79.0, I79.2, K55.1, K55.8, K55.9, Z95.8, Z95.9
Cerebrovascular disease	G45, G46, H34.0, I60 - I69, H34.0
Dementia	F00 - F03, G30, F051, G31.1
Chronic pulmonary disease	I27.8, I27.9, J40 - J47, J60 - J67, J68.4, J70.1, J70.3
Rheumatologic disease	M05, M06, M31.5, M32-M34, M35.1, M35.3, M36.0

Peptic ulcer disease	K25 - K28
Hemiplegia or paraplegia	G04.1, G11.4, G80.1, G80.2, G81, G82, G83.0 - G83.4, G83.9
Diabetes without chronic complication	E10.0, E10.1, E10.6, E10.8, E10.9, E11.0, E11.1, E11.6, E11.8, E11.9, E12.0, E12.1, E12.6, E12.8, E12.9, E13.0, E13.1, E13.6, E13.8, E13.9, E14.0, E14.1, E14.6, E14.8, E14.9
Diabetes with chronic complication	E10.2 - E10.5, E10.7, E11.2 - E11.5, E11.7, E12.2 - E12.5, E12.7, E13.2 - E13.5, E13.7, E14.2 - E14.5, E14.7
Mild liver disease	B18, K70.0 - K70.3, K70.9, K71.3 - K71.5, K71.7, K73, K74, K76.0, K76.2 - K76.4, K76.8, K76.9, Z94.4
Moderate or severe liver disease	I85.0, I85.9, I86.4, I98.2, K70.4, K71.1, K72.1, K72.9, K76.5, K76.6, K76.7
Chronic kidney disease	I12.0, I13.1, N03.2 - N03.7, N05.2 - N05.7, N18, N19, N25.0, Z49.0 - Z49.2, Z94.0, Z99.2
Malignancy, including lymphoma and leukemia, except malignant neoplasm of skin	C00 - C26, C30 - C34, C37 - C41, C43, C45 - C58, C60 - C76, C81 - C85, C88, C90 - C97
Metastatic solid tumour	C77 – C80
HIV/AIDS	B20 - B22, B24
**Hospital Frailty Risk Score**	
Dementia in Alzheimer's disease	F00
Hemiplegia	G81
Alzheimer's disease	G30
Sequelae of cerebrovascular disease (secondary codes)	I69
Other symptoms and signs involving the nervous and musculoskeletal systems (R29·6 Tendency to fall)	R29
Other disorders of urinary system (includes urinary tract infection and urinary incontinence)	N39
Delirium, not induced by alcohol and other psychoactive substances	F05
Unspecified fall	W19
Superficial injury of head	S00
Unspecified haematuria	R31
Other bacterial agents as the cause of diseases classified to other chapters (secondary code)	B96

Other symptoms and signs involving cognitive functions and awareness	R41
Abnormalities of gait and mobility	R26
Other cerebrovascular diseases	I67
Convulsions, not elsewhere classified	R56
Somnolence, stupor and coma	R40
Complications of genitourinary prosthetic devices, implants and grafts	T83
Intracranial injury	S06
Fracture of shoulder and upper arm	S42
Other disorders of fluid, electrolyte and acid-base balance	E87
Other joint disorders, not elsewhere classified	M25
Volume depletion	E86
Senility	R54
Care involving use of rehabilitation procedures	Z50
Unspecified dementia	F03
Other fall on same level	W18
Problems related to medical facilities and other health care	Z75
Vascular dementia	F01
Superficial injury of lower leg	S80
Cellulitis	L03
Blindness and low vision	H54
Deficiency of other B group vitamins	E53
Problems related to social environment	Z60
Parkinson's disease	G20
Syncope and collapse	R55

Fracture of rib(s), sternum and thoracic spine	S22
Other functional intestinal disorders	K59
Acute renal failure	N17
Decubitus ulcer	L89
Carrier of infectious disease	Z22
Streptococcus and staphylococcus as the cause of diseases classified to other chapters	B95
Ulcer of lower limb, not elsewhere classified	L97
Other symptoms and signs involving general sensations and perceptions	R44
Duodenal ulcer	K26
Hypotension	I95
Unspecified renal failure	N19
Other septicaemia	A41
Personal history of other diseases and conditions	Z87
Respiratory failure, not elsewhere classified	J96
Exposure to unspecified factor	X59
Other arthrosis	M19
Epilepsy	G40
Osteoporosis without pathological fracture	M81
Fracture of femur	S72
Fracture of lumbar spine and pelvis	S32
Other disorders of pancreatic internal secretion	E16
Abnormal results of function studies	R94
Chronic renal failure	N18
Retention of urine	R33

Unknown and unspecified causes of morbidity	R69
Other disorders of kidney and ureter, not elsewhere classified	N28
Unspecified urinary incontinence	R32
Other degenerative diseases of nervous system, not elsewhere classified	G31
Nosocomial condition	Y95
Other and unspecified injuries of head	S09
Symptoms and signs involving emotional state	R45
Transient cerebral ischaemic attacks and related syndromes	G45
Problems related to care-provider dependency	Z74
Other soft tissue disorders, not elsewhere classified	M79
Fall involving bed	W06
Open wound of head	S01
Other bacterial intestinal infections	A04
Diarrhoea and gastroenteritis of presumed infectious origin	A09
Pneumonia, organism unspecified	J18
Pneumonitis due to solids and liquids	J69
Speech disturbances, not elsewhere classified	R47
Vitamin D deficiency	E55
Artificial opening status	Z93
Gangrene, not elsewhere classified	R02
Symptoms and signs concerning food and fluid intake	R63
Other hearing loss	H91
Fall on and from stairs and steps	W10

Fall on same level from slipping, tripping and stumbling	W01
Thyrotoxicosis [hyperthyroidism]	E05
Scoliosis	M41
Dysphagia	R13
Dependence on enabling machines and devices	Z99
Agent resistant to penicillin and related antibiotics	U80
Osteoporosis with pathological fracture	M80
Other diseases of digestive system	K92
Cerebral Infarction	I63
Calculus of kidney and ureter	N20
Mental and behavioural disorders due to use of alcohol	F10
Other medical procedures as the cause of abnormal reaction of the patient	Y84
Abnormalities of heart beat	R00
Unspecified acute lower respiratory infection	J22
Problems related to life-management difficulty	Z73
Other abnormal findings of blood chemistry	R79
Personal history of risk-factors, not elsewhere classified	Z91
Open wound of forearm	S51
Depressive episode	F32
Spinal stenosis (secondary code only)	M48
Disorders of mineral metabolism	E83
Polyarthrosis	M15
Other anaemias	D64
Other local infections of skin and subcutaneous tissue	L08

Nausea and vomiting	R11
Other noninfective gastroenteritis and colitis	K52
Fever of unknown origin	R50
**Other comorbidities of interest**	
Gastro-oesophageal reflux disease	K21.9, K21.0
Anxiety disorders, mood disorders and other nonpsychotic mental disorders	F30-F48, F68
Hyperlipidaemia	E78.0 – E78.5, H02.6
Hypertension	I10, I11 - I13, I15
Hyperthyroidism	E05
Hyperparathyroidism	E21
Ischaemic heart disease	I20 – I25
Nutritional deficiencies or gastrointestinal conditions likely to result in malabsorption (Crohn's disease, celiac disease, ulcerative colitis, steatorrhea, blind loop syndrome)	E40 - E43, E44.0, E44.1, E45, E46, E50 - E54, E55.0, E55.9, E56, E58 - E61, E63, E64, K50, K51, K90.0, K90.3, K90.4, K90.8, K91.2, M83, R64
Osteoporosis	M81
Rheumatoid arthritis	M05, M06

Table S7: Explanation of the covariates used in the mortality and refracture risk analyses

Covariate	Definition
Index of relative socioeconomic disadvantage (IRSD)	The Index of Relative Socioeconomic Disadvantage (IRSD) is a general socio-economic index that summarises a range of information about the economic and social conditions of people and households within the area. All areas are ordered from lowest to highest score, then the lowest 10% of areas are given a decile number of 1, the next lowest 10% of areas are given a decile number 2 and so on, up to the highest 10% of areas which are given a decile number of 10. This means that areas are divided up into ten equal sized groups, depending on their score. A low score indicates relatively greater disadvantage in general. A high score indicates a relative lack of disadvantage in general.
Index of educational and occupational status (IEO)	The Index of Education and Occupation (IEO) is designed to reflect the educational and occupational level of communities. The education variables in this index show either the level of qualification achieved or whether further education is being undertaken. The occupation variables classify the workforce into the major groups and skill levels of the Australian and New Zealand Standard Classification of Occupations

	(ANZSCO) and the unemployed. All areas are ordered from lowest to highest score (decile number 1 to 10). A low score indicates relatively lower education and occupation status of people in the area in general. A high score indicates relatively higher education and occupation status of people in the area in general.
Prior DXA within 5 years prior to index fracture or study start	The Medicare Benefits Scheme (MBS) provides data on diagnostic imaging, including dual-energy X-ray absorptiometry (DXA) scans covered by a Medicare rebate. A DXA scan is identified by a recorded date of service within the 5-year period prior to fracture. A DXA scan indicates bone mineral density (BMD) assessment, which is directly linked to fracture risk. Additionally, individuals who undergo a DXA scan may already be at higher baseline fracture risk due to clinician concerns about osteoporosis, potentially influencing refracture likelihood.
Charlson Comorbidity Index (CCI)	The Charlson Comorbidity Index (CCI) is a validated comorbidity measure widely used in epidemiological and administrative health data studies to quantify overall disease burden and predict mortality risk. The index assigns weighted scores to a range of chronic conditions based on their association with mortality, including cardiovascular disease, chronic kidney disease, diabetes, chronic pulmonary disease, malignancy, and liver disease. Individual condition weights are summed to generate a total score, with higher scores indicating greater comorbidity burden.
Hospital Frailty Risk Score (HFRS)	The Hospital Frailty Risk Score (HFRS) is a validated frailty measure developed for use with hospital administrative data and is based on the presence of 109 ICD-10 diagnostic codes associated with frailty-related conditions. These include diagnoses reflecting cognitive impairment, falls, mobility problems, delirium, incontinence, pressure ulcers, and other markers of physiological vulnerability. Each ICD-10 diagnostic group contributes a weighted score derived from regression modelling, and the total HFRS is calculated as the sum of these weights. Higher scores indicate greater frailty risk. Consistent with the original derivation study, frailty risk was categorised as low (<5), intermediate (5–15), and high (>15).
Remoteness	The Modified Monash Model (MMM) defines whether a location is metropolitan, rural, remote or very remote. The model measures remoteness and population size on a scale of Modified Monash (MM) categories MM 1 to MM 7. MM 1 is a major city and MM 7 is very remote. Remoteness was classified as metropolitan (MM1), regional (MM2, MM3) and rural (MM4-MM7).
Polypharmacy	Concurrent use of five or more medications within 90 days before fracture
Steroid exposure	Dispensing of prednisone $\geq$5mg/day within one year before fracture
Prior hospitalisation for fall	Hospitalised fall in the five years before index fracture
Prior hospitalisations	Number of hospital admissions in the five years before index fracture

Table S8: Baseline characteristics of early initiators by drug choice

Baseline characteristic	New users – early initiators											
	Women (n=16952)						Men (n=5166)					
	Dmab (n=9027) (53.3%)	Oral N-BP (n=5812) (34.3%)	Intra-venous ZOL (n=1138) (6.7%)	Calcitriol (n=192) (1.1%)	Strontium Ranelate (n=708) (4.2%)	Raloxifene (n=75) (0.4%)	Dmab (n=2325) (45.0%)	Oral N-BP (n=2152) (41.7%)	Intra-venous ZOL (n=414) (8.0%)	Calcitriol (n=179) (3.5%)	Strontium Ranelate (n=95)	Raloxifene (n=1)
n (%)												
Comorbidities												
Anxiety/ depression	470 (5.2)	272 (4.7)	55 (4.8)	14 (7.3)	42 (5.9)	9	112 (4.8)	117 (5.4)	15 (3.6)	7 (3.9)	5	0
Cancer	596 (6.6)	308 (5.3)	80 (7.0)	19 (9.9)	37 (5.2)	4	305 (13.1)	261 (12.1)	56 (13.5)	28 (15.6)	10	0
Congestive heart failure	476 (5.3)	273 (4.7)	39 (3.4)	43 (22.4)	42 (5.9)	2	218 (9.4)	198 (9.2)	38 (9.2)	44 (24.6)	11	0
Chronic kidney disease												
- Stage 1-3	238 (2.6)	94 (1.6)	16 (1.4)	31 (16.1)	8 (1.1)	1	185 (8.0)	95 (4.4)	12 (2.9)	35 (19.6)	6	0
- Stage 4-5, dialysis	172 (1.9)	68 (1.2)	6 (0.5)	59 (30.7)	9 (1.3)	0	99 (4.3)	57 (2.6)	9 (2.2)	77 (43.0)	6	0
COPD	504 (5.6)	283 (4.9)	52 (4.6)	25 (13.0)	29 (4.1)	0	202 (8.7)	200 (9.3)	47 (11.4)	23 (12.8)	11	0
Dementia	320 (3.5)	209 (3.6)	18 (1.6)	7 (3.6)	27 (3.8)	2	110 (4.7)	129 (6.0)	12 (2.9)	7 (3.9)	8	0
Diabetes	1238 (13.7)	596 (10.3)	118 (10.4)	68 (35.4)	59 (8.3)	2	531 (22.8)	379 (17.6)	74 (17.9)	74 (41.3)	14	1

Gastro-malabsorption	72 (0.8)	40 (0.7)	10 (0.9)	0 (0)	3 (0.4)	0	23 (1.0)	9 (0.4)	3 (0.7)	1 (0.6)	1	0
GORD	682 (7.6)	280 (4.8)	113 (9.9)	15 (7.8)	46 (6.5)	2	157 (6.8)	125 (5.8)	29 (7.0)	12 (6.7)	4	0
Hyperlipidaemia	143 (1.6)	172 (3.0)	32 (2.8)	15 (7.8)	27 (3.8)	0	50 (2.2)	92 (4.3)	19 (4.6)	11 (6.1)	7	1
Hyper-parathyroidism	27 (0.3)	14 (0.2)	6 (0.5)	2 (1.0)	3 (0.4)	0	7 (0.3)	1 (0.0)	2 (0.5)	3 (1.7)	0	0
Hypertension	1552 (17.2)	959 (16.5)	194 (17.0)	85 (44.3)	146 (20.6)	10	597 (25.7)	572 (26.6)	100 (24.2)	110 (61.5)	28	0
Hyperthyroidism	105 (1.2)	58 (1.0)	11 (1.0)	8 (4.2)	8 (1.1)	0	19 (0.8)	17 (0.8)	1 (0.2)	3 (1.7)	3	0
Ischaemic heart disease	574 (6.4)	299 (5.1)	67 (5.9)	23 (12.0)	48 (6.8)	3	302 (13.0)	225 (10.5)	48 (11.6)	39 (21.8)	11	0
Chronic liver disease	68 (0.8)	50 (0.9)	12 (1.1)	9 (4.7)	4 (0.6)	1	53 (2.3)	51 (2.4)	8 (1.9)	7 (3.9)	1	0
Osteoporosis	250 (2.8)	126 (2.2)	33 (2.9)	5 (2.6)	16 (2.3)	2	155 (6.7)	118 (5.5)	29 (7.0)	16 (8.9)	3	0
Rheumatoid arthritis	65 (0.7)	28 (0.5)	14 (1.2)	0 (0)	5 (0.7)	0	11 (0.5)	14 (0.7)	6 (1.4)	1 (0.6)	0	0
Stroke	444 (4.9)	277 (4.8)	44 (3.9)	17 (8.9)	42 (5.9)	3	231 (9.9)	222 (10.3)	31 (7.5)	20 (11.2)	9	1

Abbreviations: Dmab, denosumab; N-BP, nitrogen containing bisphosphonate; ZOL, zoledronic acid SD, standard deviation; COPD, chronic obstructive pulmonary disease; GORD, gastro-oesophageal reflux disease.
